# *Wolbachia*-Mediated Antibacterial Protection and Immune Gene Regulation in *Drosophila*

**DOI:** 10.1371/journal.pone.0025430

**Published:** 2011-09-29

**Authors:** Zhee Sheen Wong, Lauren M. Hedges, Jeremy C. Brownlie, Karyn N. Johnson

**Affiliations:** 1 School of Biological Sciences, The University of Queensland, Brisbane, Queensland, Australia; 2 School of Biomolecular and Physical Sciences, Griffith University, Brisbane, Queensland, Australia; Indian Institute of Science, India

## Abstract

The outcome of microbial infection of insects is dependent not only on interactions between the host and pathogen, but also on the interactions between microbes that co-infect the host. Recently the maternally inherited endosymbiotic bacteria *Wolbachia* has been shown to protect insects from a range of microbial and eukaryotic pathogens. Mosquitoes experimentally infected with *Wolbachia* have upregulated immune responses and are protected from a number of pathogens including viruses, bacteria, *Plasmodium* and filarial nematodes. It has been hypothesised that immune upregulation underpins *Wolbachia*-mediated protection. *Drosophila* is a strong model for understanding host-*Wolbachia*-pathogen interactions. *Wolbachia*-mediated antiviral protection in *Drosophila* has been demonstrated for a number of different *Wolbachia* strains. In this study we investigate whether *Wolbachia*-infected flies are also protected against pathogenic bacteria. *Drosophila simulans* lines infected with five different *Wolbachia* strains were challenged with the pathogenic bacteria *Pseudomonas aeruginosa* PA01, *Serratia marcescens* and *Erwinia carotovora* and mortality compared to paired lines without *Wolbachia*. No difference in mortality was observed in the flies with or without *Wolbachia*. Similarly no antibacterial protection was observed for *D. melanogaster* infected with *Wolbachia*. Interestingly, *D. melanogaster* Oregon RC flies which are naturally infected with *Wolbachia* showed no upregulation of the antibacterial immune genes TepIV, Defensin, Diptericin B, PGRP-SD, Cecropin A1 and Attacin D compared to paired flies without *Wolbachia*. Taken together these results indicate that *Wolbachia*-mediated antibacterial protection is not ubiquitous in insects and furthermore that the mechanisms of antibacterial and antiviral protection are independent. We suggest that the immune priming and antibacterial protection observed in *Wolbachia*-infected mosquitoes may be a consequence of the recent artificial introduction of the symbiont into insects that normally do not carry *Wolbachia* and that antibacterial protection is unlikely to be found in insects carrying long-term *Wolbachia* infections.

## Introduction

The interaction between two microbes within a host can impact on the outcome of infection for the host. *Wolbachia* are a maternally transmitted endosymbiotic α-proteobacteria that is predicted to infect up to 70% of insect species [1], [2]. *Wolbachia* can protect insects from infection by a range of microbes and parasites [3]–[10]. Where the microbes are pathogens of the insect, this protection has the potential to greatly influence the ecology of the host, pathogen and *Wolbachia* [11]–[13]. In addition, it has been widely suggested that *Wolbachia*-mediated pathogen protection could be harnessed in biological control programs to interfere with the transmission of human diseases that are vectored by insects, including dengue and malaria. However, the molecular mechanisms involved in protection are yet to be determined.

Mosquitoes that are experimentally infected with *Wolbachia* are protected from a range of viruses, bacteria and parasites. A number of mosquito species that are important human disease vectors are not naturally found to be infected with *Wolbachia*, for example *Aedes aegypti* and *Anopheles* species. However, utilising transinfection techniques *Ae. aegypti* has been experimentally infected with *Wolbachia* strains *w*MelPop-CLA or *w*AlbB [14], [15]. The mosquitoes stably infected with *Wolbachia* accumulate and transmit RNA viruses such as Dengue and Chikungunya less readily than *Wolbachia*-free mosquitoes [3], [8]. Infection of *Ae. aegypti* with the *D. melanogaster* derived *Wolbachia* strain *w*MelPop-CLA also reduced the prevalence of the filarial nematode *Brugia pahangi*, impaired the ability to transmit the avian malarial parasite *Plasmodium gallinaceum*. In addition reduced mortality induced by infection with the Gram-negative bacterium *Erwinia caratovora* but not the Gram-positive bacteria *Micrococcus luteus* was observed in these mosquitoes [7]. In addition, whilst stable transinfection of *Anopheles* mosquitoes is yet to be achieved, *An. gambiae* that were somatically infected with *Wolbachia* showed reduced accumulation of *Plasmodium* oocysts [5], [6]. Thus in mosquitoes, artificially introduced *Wolbachia* induces broad ranging antipathogen protection.

The phenomenon of *Wolbachia*-mediated antiviral protection is well established in the model insect *Drosophila*. Naturally *Wolbachia*-infected flies are protected from a diverse range of RNA viruses [4], [10]. In the case of the pathogenic viruses *Drosophila C virus* (DCV), *Flock House virus* (FHV) and *Cricket paralysis virus* (CrPV) *Wolbachia*-infected flies survive upwards of twice as long as their unprotected *Wolbachia*-free counterparts [4], [10]. This protection has been shown to be consistent across the closely related *Wolbachia* strains that infect *D. melanogaster* (*w*Mel, *w*MelCS and *w*MelPop) and across different host backgrounds [4], [10]. *Wolbachia* antiviral protection has also been demonstrated in the related species *D. simulans* [9].

Not all *Wolbachia*∶host combinations result in antiviral protection. *D. simulans* are naturally infected with diverse *Wolbachia* strains from both supergroup A (*w*Au, *w*Ri and *w*Ha) and B (*w*No) [16], [17]. *D. simulans* lines naturally infected with *w*Au and *w*Ri (line CO and DSR respectively) are protected from DCV and FHV, whereas those naturally infected with *w*Ha and *w*No (line DSH and N7NO respectively) are not protected [9]. In these fly lines antiviral protection correlates with both phylogenetic relatedness to *w*Mel and also high density of *Wolbachia* in the host [9]. The *D. simulans* line Me29, which was transinfected with *w*Mel in 1998 [18], is also protected from both DCV and FHV infection.

The mechanism(s) of *Wolbachia*-mediated protection have not been determined. The correlation between density and distribution of *Wolbachia* in flies and mosquitoes supports the hypothesis that *Wolbachia* and pathogens may be in competition for limited host resources [8], [9]. Alternatively several studies have demonstrated that antipathogen protection in experimentally infected mosquitoes is concomitant with *Wolbachia* induced upregulation of a range of host immune genes [3], [5]–[8]. Genes involved in the antimicrobial IMD and Toll pathways are upregulated, with the effector genes such as cecropins and other antimicrobial peptides (AMPs) showing the highest upregulation. These observations led to the hypothesis that *Wolbachia* infection primes the immune system so that when *Wolbachia*-infected insects are challenged with a pathogen the insect is protected from the pathogen. Little direct evidence is available in support of this hypothesis, although in somatically infected *An. gambiae* upregulation of Tep1 has been experimentally linked with protection against parasite *P. berghei* [6] and Dengue virus is somewhat impacted by upregulation of the Toll pathway [19].

In contrast to mosquitoes, it is less clear if *Wolbachia* stimulates immune priming in *Drosophila*. Cultured *D. melanogaster* cells (S2 cell line) showed upregulation of genes from the Toll and IMD pathways as well as AMP effector molecules when experimentally infected with the *Wolbachia w*Ri strain (which naturally infects *D. simulans*) [20]. In contrast, an early study using Northern blot analysis found no difference in cecropin or diptericin RNA levels in *D. simulans* line DSR with and without *w*Ri infection and similarly no difference was recorded for Defensin expression in *Ae. albopictus* with and without *Wolbachia* infection [21]. It remains to be confirmed whether *Wolbachia*-mediated immune priming is linked to the antiviral protection that has been documented in *Drosophila*.

In mosquitoes *Wolbachia* mediates protection against a range of pathogens, yet it is unclear whether a single molecular mechanism underpins protection against this diverse group of microbes and parasites. In *Drosophila* not all host∶*Wolbachia* combinations protect against virus infection [9]. If the mechanism that underlies protection were the same for bacteria and viruses we would predict that those *Drosophila*∶*Wolbachia* combinations with reduced virus-induced mortality would similarly reduce pathogenic bacterial infection.

In this study we investigated whether antibacterial protection occurs in flies infected with *Wolbachia*. To do this five *D. simulans*∶*Wolbachia* lines were utilised that we previously used to investigate antiviral protection. Bacterial pathogens *Pseudomonas aeruginosa* PA01, *Serratia marcescens* and *E. carotovora* were used to challenge the flies. *P. aeruginosa* PA01 and *S. marcescens* are the opportunistic and natural pathogens of *Drosophila* respectively [22], [23] and *Wolbachia* mediates protection in *Ae. aegypti* against mortality induced by *E. carotovora* [7]. Further, using reverse transcription and quantitative PCR to assay expression of six AMPs and immune genes there was no evidence of antibacterial immune priming in *D. melanogaster* naturally infected with *Wolbachia*.

## Results

### *Wolbachia* does not protect *D. simulans* from pathogenic bacteria

The impact of *Wolbachia* on the outcome of virus infection varies in *D. simulans* lines challenged with RNA viruses. *D. simulans* lines CO, DSR and Me29 are protected against DCV and FHV infections by *Wolbachia* strains *w*Au, *w*Ri and *w*Mel respectively. In contrast, *D. simulans* lines DSH and N7NO are not protected against DCV and FHV infections by *w*Ha and *w*No respectively [9]. In order to investigate whether these *Wolbachia* strains confer protection to *D. simulans* lines challenged with pathogenic bacteria, *D. simulans* CO, DSR, Me29, DSH and N7NO lines with *Wolbachia* and paired lines that had been cured of *Wolbachia* infection were challenged with three pathogenic Gram-negative bacteria (*P. aeruginosa* PA01, *S. marcescens* and *E. carotovora*) and mortality recorded for up to 36 hours.

**Figure 1:**
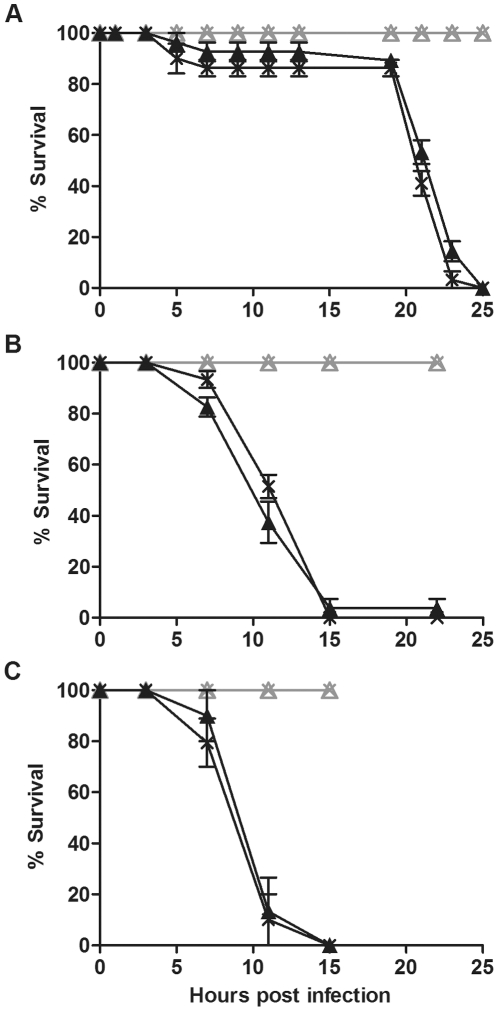
Survival of *D. simulans* CO flies challenged with pathogenic bacteria. Graphs show the survival of *D. simulans* CO flies with (cross) and without (triangle) *w*Au challenged with (A) *P. aeruginosa* PA01, (B) *S. marcescens* and (C) *E. carotovora*. Flies were infected with pathogenic bacteria (black line) or mock infected with LB (grey line). Error bars represent SEM calculated from three replicate vials.

Mortality of CO flies challenged with pathogenic bacteria was similar regardless of *Wolbachia* infection status (Figure 1). Flies both with and without *Wolbachia* challenged with *P. aeruginosa* PA01 died within 25 hours of infection and there was no significant difference in the survival curves (Figure 1 A; p  =  0.2). In this and all other experiments there was negligible mortality of mock-infected flies during the time course. *S. marcescens* and *E. carotovora* are more virulent than *P. aeruginosa* PA01. After infection, flies with and without *Wolbachia* died within 15 hours (Figure 1 B and C). Statistical analysis showed that there was no significant difference in survival of flies with and without *Wolbachia* (p  =  0.3 for *S. marcescens* infection, p  =  0.0533 for *E. carotovora* infection). Each survival bioassay was independently repeated at least three times with similar results (data not shown). These results indicate that while *w*Au infection of CO flies protects the flies from viral-induced mortality there is no protection against pathogenic bacteria mediated by *w*Au.

**Figure 2:**
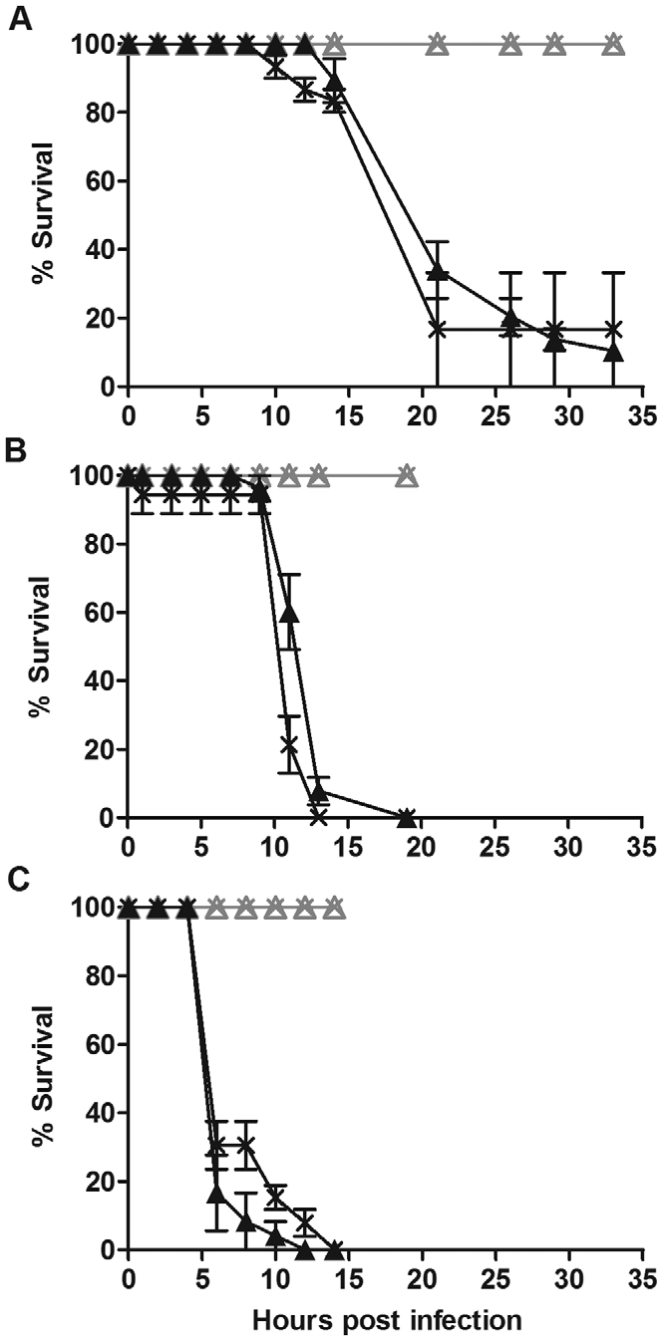
Survival of *D. simulans* DSR flies challenged with pathogenic bacteria. Graphs show the survival of *D. simulans* DSR flies with (cross) and without (triangle) *w*Ri challenged with (A) *P. aeruginosa* PA01, (B) *S. marcescens* and (C) *E. carotovora*. Flies were infected with pathogenic bacteria (black line) or mock infected with LB (grey line). Error bars represent SEM calculated from three replicate vials.

**Figure 3:**
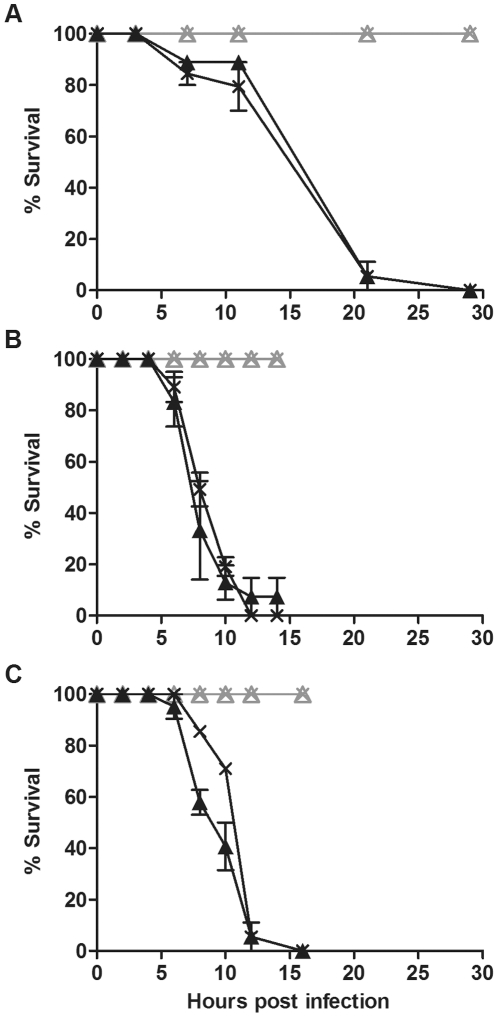
Survival of *D. simulans* Me29 flies challenged with pathogenic bacteria. Graphs show the survival of *D. simulans* Me29 with (cross) and without (triangle) *w*Mel challenged with (A) *P. aeruginosa* PA01, (B) *S. marcescens* and (C) *E. carotovora*. Flies were infected with pathogenic bacteria (black line) or mock infected with LB (grey line). Error bars represent SEM calculated from three replicate vials.

**Figure 4:**
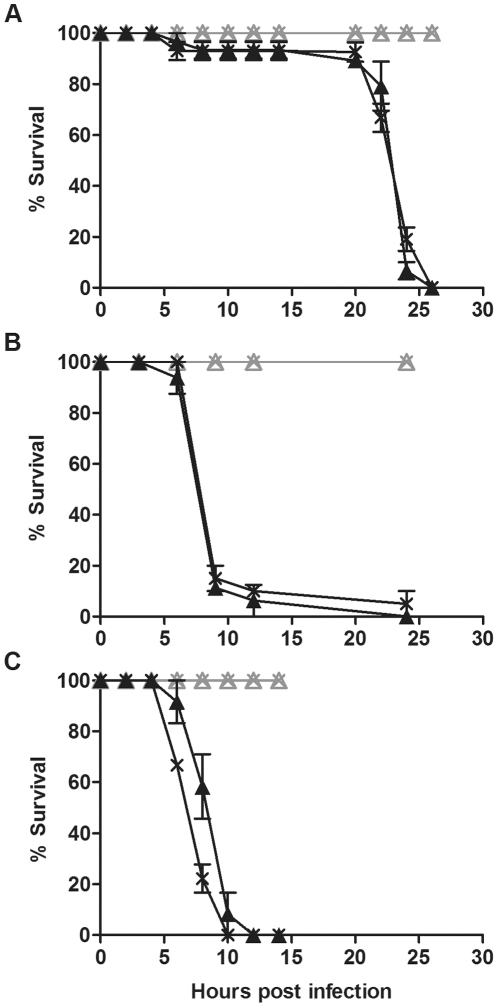
Survival of *D. simulans* DSH flies challenged with pathogenic bacteria. Graphs show the survival of *D. simulans* DSH infected with (cross) and without (triangle) *w*Ha challenged with (A) *P. aeruginosa* PA01, (B) *S. marcescens* and (C) *E. carotovora*. Flies were infected with pathogenic bacteria (black line) or mock infected with LB (grey line). Error bars represent SEM calculated from three replicate vials.

**Figure 5:**
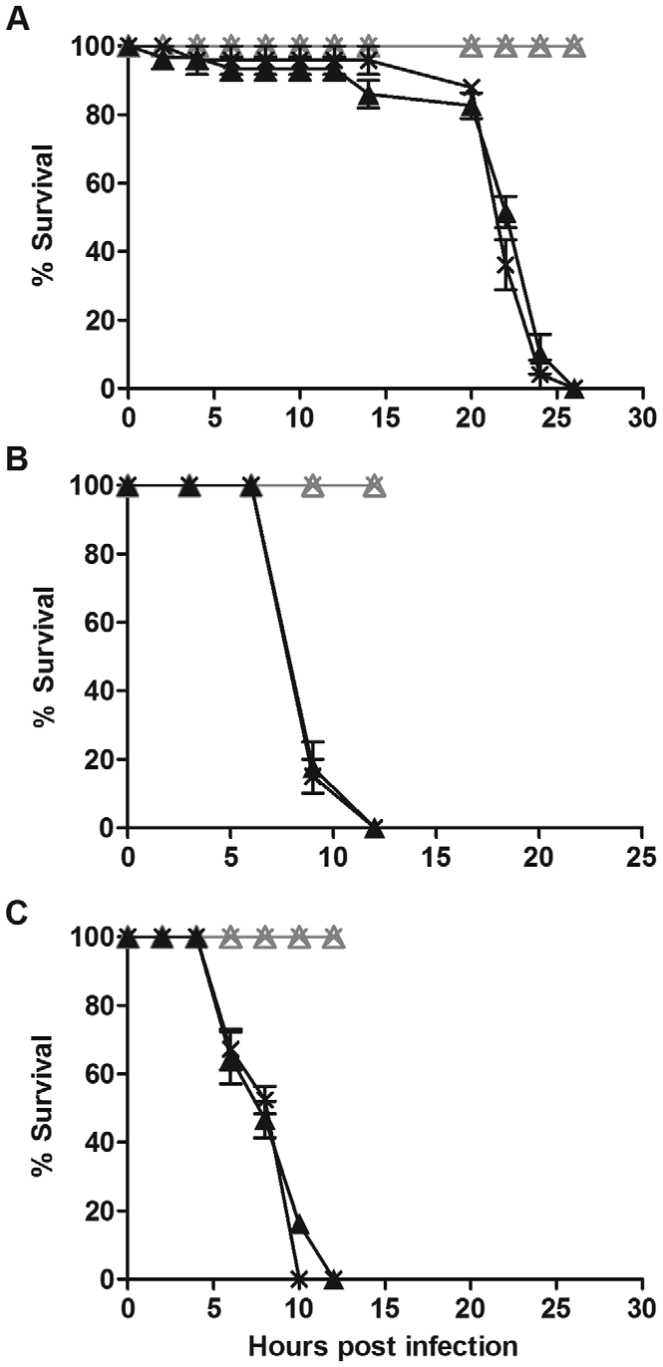
Survival of *D. simulans* N7NO challenged with pathogenic bacteria. Graphs show the survival of *D. simulans* N7NO with (cross) and without (triangle) *w*No challenged with (A) *P. aeruginosa* PA01, (B) *S. marcescens* and (C) *E. carotovora*. Flies were infected with pathogenic bacteria (black line) or mock infected with LB (grey line). Error bars represent SEM calculated from three replicate vials.

To investigate whether lack of protection in *D. simulans* was limited to this particular host∶*Wolbachia* combination we challenged four other fly lines with and without *Wolbachia* with the three pathogenic bacteria. DSR, Me29, DSH and N7NO that were challenged with *P.aeruginosa* PA01 died within 25 to 30 hours post infection (Figure 2 A, 3 A, 4 A and 5 A). *D. simulans* DSR, Me29, DSH and N7NO when challenged with *S. marcescens* and *E. carotovora*, died within 10 to 25 hours post infection (Figure 2 B and C, 3 B and C, 4 B and C, 5 B and C). In each assay there was no difference between the survival curves of flies with and without *Wolbachia* challenged with each of the pathogenic bacteria (p>0.05). Results shown are representative of at least three independent bioassays, each with similar results obtained. The survival bioassays were also repeated with at least two independent experiments with a lower concentration of bacterial culture (O.D._600 nm_  =  0.1–0.5) (data not shown). At this lower concentration of bacteria, 100% mortality was not achieved in most cases, however, there was still no protection against bacterial-induced mortality. Taken together these results give strong evidence that *Wolbachia* does not confer protection in *D. simulans* against bacterial-induced mortality.

### Analysis of *Wolbachia*-mediated antibacterial protection in *D. melanogaster*

**Figure 6:**
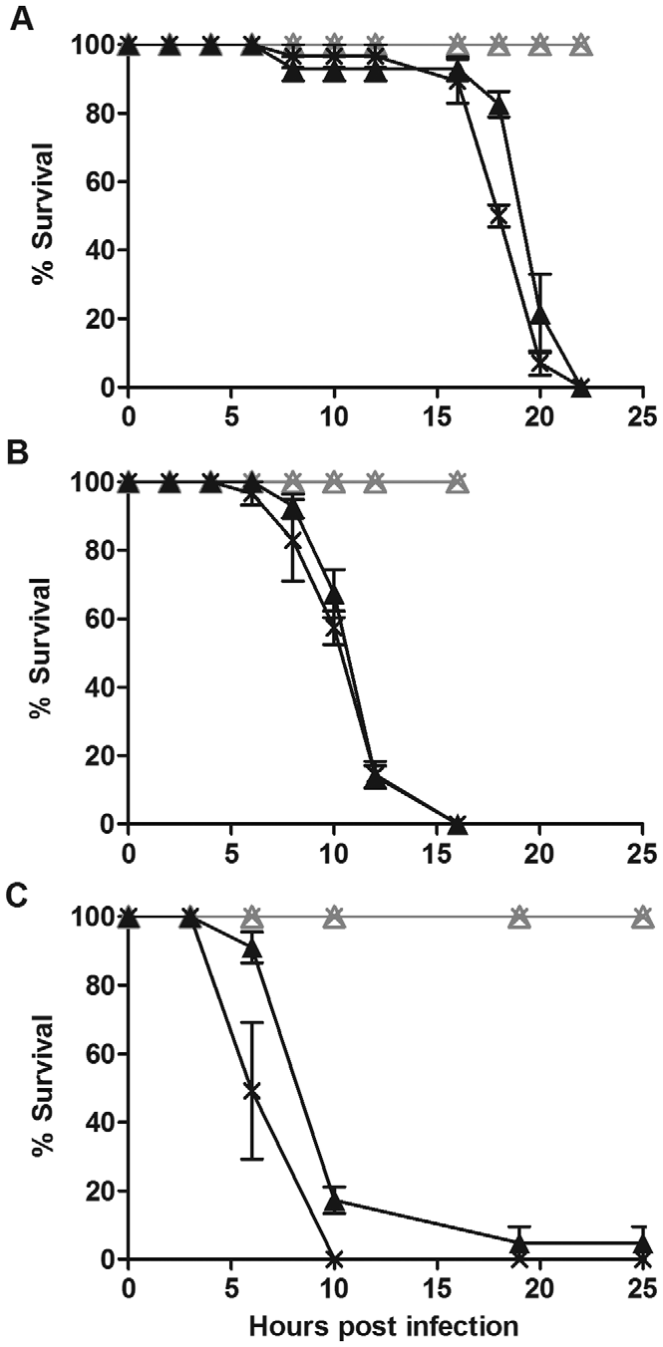
Survival of *D. melanogaster* Oregon RC challenged with pathogenic bacteria. Graphs show the survival of *D. melanogaster* ORC with (cross) and without (triangle) *w*MelCS challenged with (A) *P. aeruginosa* PA01, (B) *S. marcescens* and (C) *E. carotovora*. Flies were infected with pathogenic bacteria (black line) or mock infected with LB (grey line). Error bars represent SEM calculated from three replicate vials.

In contrast to *Ae. aegypti* mosquitoes [7] our results show that there is no antibacterial protection mediated by *Wolbachia* in *D. simulans*. To investigate whether lack of protection was restricted to this single species we utilised the *D. melanogaster* line ORC in protection bioassays. This species was chosen as *Wolbachia* has been shown to protect *D. melanogaster* from a number of different viral pathogens [4], [9], [10]. ORC flies with and without *w*MelCS were challenged with *P. aeruginosa* PA01, *S. marcescens* and *E. carotovora* and survival was monitored (Figure 6). There was no difference in the survival of flies with and without *Wolbachia* in response to *S. marcescens* challenge (p  =  0.3). ORC flies with *Wolbachia* were somewhat more susceptible to *P. aeruginosa* PA01 and *E. carotovora* than flies without *Wolbachia* (p<0.05) in the results shown in Figure 6. However, this small difference was only observed in one out of three experiments, suggesting that if there is a biological difference it is negligible. These results show that natural infection with *Wolbachia* does not confer protection against pathogenic bacteria-induced mortality in *D. melanogaster*.

### Analysis of *in vivo* bacterial growth in *D. melanogaster*

**Figure 7:**
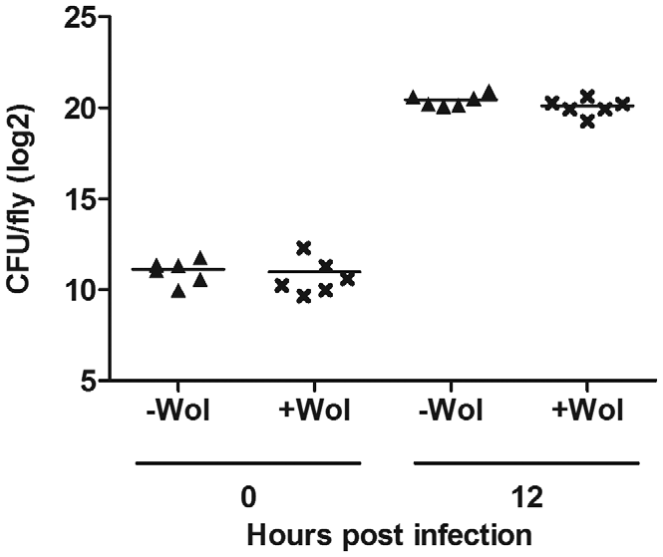
*In vivo* bacterial growth of *P. aeruginosa* PA01 *D. melanogaster* ORC adults with and without *Wolbachia* infection. Graph shows the number of bacteria per fly (CFU/fly) of *P. aeruginosa* PA01 in *D. melanogaster* without (triangle) and with *w*MelCS (cross) at time 0 and 12 hours post infection.

Taken together, results obtained from survival bioassays of all *D. simulans* and *D. melanogaster* challenged with pathogenic bacteria indicate that *Wolbachia* does not mediate protection against mortality induced by pathogenic bacteria in *Drosophila*. In order to investigate whether *Wolbachia* affects the *in vivo* accumulation of pathogenic bacterial load in *Drosophila* the *D. melanogaster* ORC line was challenged with *P. aeruginosa* PA01. Samples were collected at 0 and 12 hours post infection and bacterial load was analysed. In flies that were not challenged with *P. aeruginosa* PA01 no bacterial colonies grew on the selective LB-Ampicillin plates, indicating that all colonies identified in challenged flies arose from antibiotic resistant *P. aeruginosa* PA01 bacteria. At time 0 bacterial counts were 1.0–3.5×10^3^ CFU/fly, indicating that this was the dose with which the flies were challenged (Figure 7). At 12 hours post infection the bacterial load had increased by 3 orders of magnitude to approximately 1.0×10^6^ CFU/fly. Similar bacterial loads were observed in both flies with and without *Wolbachia* indicating that there was no difference in the accumulation of the *P. aeruginosa* PA01.

### Regulation of immune genes in *D. melanogaster* in response to *Wolbachia* infection

**Figure 8:**
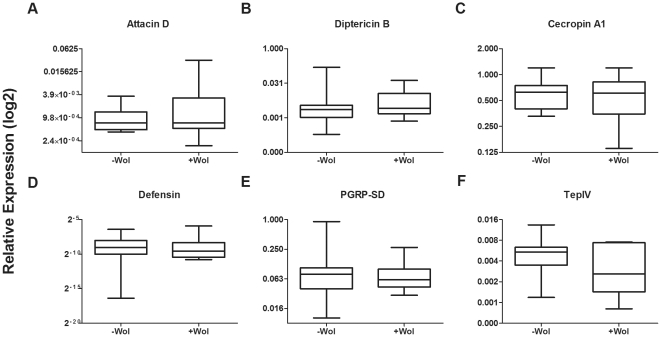
Immune gene expression in response to *Wolbachia* infection. The relative expression (RE) of immune genes of *D. melanogaster* with and without *Wolbachia* was analysed using RT-qPCR. The box and whisker plots show the ratio of immune to reference gene (RpL32) expression from the indicated genes: (A) Attacin D, (B) Diptericin B, (C) Cecropin A1, (D) Defensin, (E) PGRP-SD and (F) TepIV. Boxes represent medians, 25 (bar below median) and 75 (above median) percentiles of 10 individual male flies. None of the medians were significantly different (Mann Whitney-U test; p>0.05).

*Wolbachia* has been shown to stimulate different immune gene responses in different *Wolbachia*∶host combinations [6]–[8], [20], [21], [24], [25] and it has been suggested that immune priming stimulated by *Wolbachia* may be causally linked to *Wolbachia*-mediated antipathogen protection [3], [6]–[8]. Given no antibacterial protection was mediated in the six host∶*Wolbachia* combinations utilised in this study we wanted to investigate whether antibacterial immune genes were upregulated by the presence of *Wolbachia* in our system. The regulation of six immune genes in *D. melanogaster* ORC flies with and without *Wolbachia* was investigated using RT-qPCR. These genes were chosen as they were homologous to genes that were upregulated by *Wolbachia* presence in *Ae. aegypti* [7]. None of the six immune genes that were investigated in *Drosophila* (TepIV, Defensin, Diptericin B, PGRP-SD, Cecropin A1 and Attacin D) were differentially regulated in the presence of *Wolbachia* (p<0.05; Figure 8).

## Discussion

The lack of *Wolbachia*-mediated antibacterial protection in *Drosophila* differs from studies in mosquitoes where *Wolbachia* has been shown to mediate broad-spectrum anti-microbe and -parasite protection [3], [5]–[8]. In mosquitoes experimentally infected with *Wolbachia* there is upregulation of a number of genes for immune effector molecules and those involved in antimicrobial pathways [3], [7], [8]. It has been suggested that this *Wolbachia* induced “immune priming” may be the mechanism underlying protection and some evidence has been presented for this for anti-*Plasmodium* protection [6]. We reasoned that as *Wolbachia*-infected *Drosophila* were not protected from pathogenic Gram-negative bacteria, this may be because *Wolbachia* was not stimulating an immune response in these flies. To assess this we analysed the expression of six antimicrobial immune genes whose homologues were upregulated in *Wolbachia*-infected mosquitoes [7]. In *D. melanogaster Wolbachia* infection did not stimulate expression of the antimicrobial response genes.

It is interesting that *Wolbachia* differentially stimulates immune responses in different hosts. The host∶*Wolbachia* examples discussed above differ in two ways. Firstly, the hosts are from different insect families: mosquitoes and flies. But perhaps more importantly each analysis of immune regulation has been performed using mosquitoes that have been recently experimentally infected with *Wolbachia*. In contrast the *D. melanogaster* ORC line used here, is naturally infected with *Wolbachia*. As a maternally inherited endosymbiont, *Wolbachia* remains in close association with its host from generation to generation. Co-evolution of the bacteria and host is postulated to eventuate in commensal or mutualistic associations. Experimental introduction of *Wolbachia* into a new host can lead to over-replication of the bacteria and pathogenicity, although these effects can be ameliorated in later generations [5], [26], [27]. This raises the possibility that *Wolbachia* induced immune priming is a consequence of maladapted interactions following experimental introduction of *Wolbachia* into a new host. This premise is supported by previous studies that show upregulation of immune genes in *D. melanogaster* cell line S2 experimentally infected with *D. simulans* derived *Wolbachia* strain wRi [20], but not in *D. simulans* flies which were naturally infected with wRi or *Ae. albopictus* naturally infected with wAlbB [21]. Further some natural *Wolbachia* infections can depress antibacterial immunity [25]. It should be noted that the *D. simulans* Me29 line used in the present study is artificially infected with the *D. melanogaster* derived *Wolbachia* strain *w*Mel. We suggest that the lack of antibacterial protection in the Me29 line may be a consequence of adaptation that has occurred during the 13 or more years since this line was established [18]. Thus both the data presented here and previously is consistent with *Wolbachia*-mediated immune priming being important for antibacterial and antiparasite protection in hosts artificially infected with *Wolbachia*.

This study investigated *Wolbachia*-mediated antibacterial protection by utilising three Gram-negative bacterial challenge models. It is possible that the protection response upon challenge with a Gram-positive bacterium may differ from that observed in this study. However, we consider this to be unlikely given the lack of protection against Gram-positive bacteria in mosquitoes [7] and lack of *Wolbachia*-mediated immune stimulation observed in naturally *Wolbachia*-infected *Drosophila*.

The immune pathways Toll, IMD and AMPs showed no evidence of antibacterial immune priming by *Wolbachia* in *D. simulans* [21] or *D. melanogaster*, and no protection against bacterial infection was observed. Given that antiviral protection has been demonstrated using the same *Drosophila* lines as used in the current study [4], [9], our results indicate that stimulation of the Toll, IMD or AMPs pathways are not necessary for *Wolbachia* stimulated antiviral mechanisms. This leaves other immune pathways, such as Vago and vir-1 [28], [29], the major insect viral-defence RNA silencing pathway [30] or competition for host resources as potential *Wolbachia*-mediated antiviral responses. As *Wolbachia*-mediated antiviral protection occurs in both naturally and experimentally infected hosts [3], [4], [8]–[10], [31] its likely to occur through a conserved mechanism that is independent of that involved in protection against other pathogens.

*Wolbachia*-mediated antiviral protection in *Drosophila* has been shown to be robust. Antiviral protection is observed against many different RNA viruses including both natural pathogens of *Drosophila* and viruses that normally infect mosquito vectors or other insects [4], [9], [10], [32]. In addition, several different *Wolbachia* strains have been shown to protect flies from viruses in a number of different lines of both *D. simulans* and *D. melanogaster* [4], [9]. In stark contrast, we show here that *Wolbachia* does not mediate protection against pathogenic bacteria in *Drosophila*. We have demonstrated this using three different Gram-negative bacterial pathogens and six different *Wolbachia*∶*Drosophila* combinations, including both *D. simulans* and *D. melanogaster* hosts. These results have implications both for the potential mechanisms of and uses for *Wolbachia*-mediated protection.

## Materials and Methods

### Flies and *Wolbachia*

All fly lines were maintained on standard cornmeal diet at 25°C with 12-hours light/dark cycle and were sourced from the lab fly collection. The *D. melanogaster* line Oregon RC (ORC) is naturally infected with *Wolbachia* strain *w*MelCS [33]. The *D. simulans* lines CO, DSR, N7NO and DSH are naturally infected with *Wolbachia* strains *w*Au [34], *w*Ri [35], *w*No [36] and *w*Ha [37] respectively. The *D. simulans* line Me29 was experimentally infected with *w*Mel over a decade ago by transinfection of NAaTC embryos with *w*Mel from *D. melanogaster* embryos [18]. *Wolbachia*-free lines were generated from each of the fly lines as previously described [4], [9].

### Pathogenic bacteria

Three Gram-negative bacteria that are pathogens of *Drosophila* were used in challenge experiments. *S. marcescens* and *E. carotovora* were grown in LB medium [23]. *P. aeruginosa* PA01 carries an ampicillin resistance gene and was grown in LB medium supplemented with 100 µg/ml ampicillin [38]. To prepare bacteria for challenge bioassays, LB broth was inoculated with bacteria from single colonies on agar plates and incubated for 16 hours at 37°C. Bacteria were then pelleted by centrifugation at 1,500× g for 10 minutes at room temperature. Fresh bacterial pellets were prepared for each infection bioassay.

### Survival bioassays

To analyse the susceptibility of flies with and without *Wolbachia* to bacterial induced mortality, 4–7 day old adult male *Drosophila* were challenged with each of the pathogenic bacteria. Flies were anaesthetised with CO_2_ prior to infection. A thin needle (diameter  =  0.193 mm) was dipped into the undiluted bacterial pellet and used to prick into the thoracic region of each fly. For each fly line, two groups of flies were challenged with pathogenic bacteria: flies with *Wolbachia* and flies without *Wolbachia*. For each group of flies three vials of ten flies were challenged with one of the pathogenic bacteria and one vial of ten flies was mock infected with LB medium. Following challenge flies were maintained in a 25°C incubator and survival of the flies was monitored every 2–6 hours. Mortality within the first 6 hours was deemed to be due to needle injury. At least three independent survival bioassays were done for each bacteria/fly line combination. Survival curves of the two groups of flies were compared using Kaplan-Meier analysis and log-rank statistics reported (GraphPad Prism).

### *In vivo* bacterial growth

To analyse the impact of *Wolbachia* on the accumulation of pathogenic bacteria in flies, 4–7 day old adult male ORC flies with and without *Wolbachia* were infected with *P. aeruginosa* PA01 as described above. At 0 and 12 hpi, three live flies were collected individually into 1.5 ml tubes. After addition of 200 µl of LB medium supplemented with 100 µg/ml ampicillin and two 3 mm glass beads (Sigma-Aldrich) to each individual, flies were homogenised for 90 s using a TissueLyser II (Qiagen). Fly homogenates were serially diluted and spread on LB agar plates containing 100 µg/ml ampicillin. Colony forming units per fly (CFU/fly) were calculated after overnight incubation of plates. The experiment was replicated on two independent cohorts of flies and the data pooled.

### Analysis of immune gene regulation

RT-qPCR was used to compare the expression of six immune genes in ORC flies with and without *Wolbachia*. Genes were chosen on the basis of homology to genes that were upregulated in *Ae. aegypti* in the presence of *Wolbachia* [7]. Primer sets for the target genes (Table S1) were designed with Primer3 software [39]. The primer efficiency across a six 5-fold cDNA dilution series was confirmed [40].

For comparison of gene regulation five individual 4–7 day old male flies from the ORC flies with and without *Wolbachia* were frozen and homogenised in Ribozol (Amesco) with two 3 mm glass beads using TissueLyser II (Qiagen) for 90 seconds with the frequency of 30 shakes/s. Total RNA was extracted and treated with DNase (Promega) for 30 minutes at 37°C to eliminate DNA contamination. 1 µg of total RNA was reverse transcribed using random primers (Promega) and SuperScript III reverse transcriptase (Invitrogen) as per the manufacturer's protocol. Quantitative PCR was performed in duplicate reactions using Platinum SYBR® green qPCR supermix as per manufacturers instructions (Invitrogen). The temperature profile for the qPCR was 95°C for 2 min, 50°C for 2 min and 40 cycles of 95°C for 10 s, 60°C for 10 s and 72°C for 20 s. qPCR was performed using a Rotor-Gene 6000 (Qiagen). Expression of the target genes was normalised using reference genes Actin 79b (GenBank accession no. NM_079486) and ribosomal protein L 32 (RpL 32) (Genbank accession no. NM_001144656.2) [41]. Target to reference gene ratios were obtained suing QGene4.2 [42] and treatment effects on the expression ratios were assessed using Mann Whitney-U tests in STATISTICA V8 (StatSoft).

## Supporting Information

Table S1 RT-qPCR primers.
(DOCX)
