# Supplementary material for: Wolbachia-Mediated Antibacterial Protection and Immune Gene Regulation in Drosophila
Source: PLoS One. 2011 Sep 29;6(9):e25430. doi: 10.1371/journal.pone.0025430 (PMC3183045; doi:10.1371/journal.pone.0025430)
Supplement: Table S1 — RT-qPCR primers. (DOCX) [file pone.0025430.s001.docx]

**Table S1. RT-qPCR primers.**

| Gene | FlyBase (Genbank) ID | Forward Primer (5’-3’) | Reverse Primer (5’-3’) | Product Size |
| --- | --- | --- | --- | --- |
| Thiolester containing protein IV | FBgn0041180  (NM_078879.2) | AGCAGGTTTCAGTGCGAGAT | AACTGGCGAAGTCCTTGAGA | 237 |
| Defensin | FBgn0010385  (NM_078948.3) | GTTCTTCGTTCTCGTGGCTATC | CTCGCTTCTGGCGGCTAT | 152 |
| Diptericin B | FBgn0034407  (NM_079063.3) | CTGATCCCCGAGAGATTGTG | GCTCAGATCGAATCCTTGCT | 143 |
| PGRP-SD | FBgn0035806  (NM_139888.2) | ATGACTTGGATCGGTTTGCT | GTAACATCATCCGCACAAGC | 185 |
| Cecropin A1 | FBgn0000276  (NM_079849.3) | GTCGCTCAGACCTCACTGC | CGACGAAAACGAAGATGTTG | 100 |
| Attacin D | FBgn0038530  (NM_079667.2) | AGTGGGGGTCACTAGGGTTC | GTGGCGTTGAGGTTGAGATT | 105 |
| Actin 79b | FBgn0000045  (NM_079486) | CCGCAAGGATCTGTATGCCA | ACGGAGTACTTGCGCTCTGG | 150 |
| RpL 32 | FBgb0002626  (NM_001144656.2) | GACGCTTCAAGGGACAGTATCTG | AAACGCGGTTCTGCATGAG | 144 |
